# Reduced intra-frontal functional connectivity during a verbal fluency task in depressed individuals with autistic traits: an fNIRS study

**DOI:** 10.3389/fnhum.2026.1778517

**Published:** 2026-08-27

**Authors:** Michihiko Koeda, En Hosokawa, Akihiro Kumagai, Yuki Ishikawa, Kento Date, Yasushi Kyutoku, Kengo Shimoda, Mahito Kimura, Ippeita Dan

**Affiliations:** 1 Department of Neuropsychiatry, Nippon Medical School Tama Nagayama Hospital, Tokyo, Japan; 2 Department of Neuropsychiatry, Graduate School of Medicine, Nippon Medical School, Tokyo, Japan; 3 Applied Cognitive Neuroscience Laboratory, Faculty of Science and Engineering, Chuo University, Tokyo, Japan; 4 Faculty of Global Management, Chuo University, Tokyo, Japan; 5 Department of Mental Health, Nippon Medical School Chiba Hokusoh Hospital, Chiba, Japan

**Keywords:** autistic traits, depressive symptoms, fNIRS, functional connectivity, prefrontal cortex, verbal fluency task

## Abstract

**Background:**

Depressive symptoms accompanied by autistic traits represent an important source of clinical heterogeneity. While aberrant functional connectivity (FC) within prefrontal networks has been implicated as a shared neural feature of both autism spectrum disorder (ASD) and depression, task-evoked FC abnormalities in individuals experiencing both remain poorly understood. This study employed functional near-infrared spectroscopy (fNIRS) to evaluate task-evoked FC during a verbal fluency task (VFT), with a focus on prefrontal network organization.

**Methods:**

Fifty neurotypical controls and 43 individuals experiencing depressive symptoms were enrolled. The depressive-state cohort was further subdivided into high autistic traits (DP_AQ-high) and low autistic traits (DP_AQ-low) subgroups based on Autism-Spectrum Quotient (AQ) scores. FC among channels in frontal, temporal, and inferior parietal regions was calculated using zero-lag Pearson correlation analysis of oxyhemoglobin (HbO₂) and deoxyhemoglobin (HbR) signals during the VFT. Between-group FC differences were evaluated using ANOVA for the Control versus depressive-state comparison, and medication-adjusted ANCOVA for the DP_AQ-high versus DP_AQ-low comparison, with diazepam-equivalent anxiolytic dosage and imipramine-equivalent antidepressant dosage as covariates.

**Results:**

Relative to neurotypical controls, the depressive-state group exhibited decreased interhemispheric frontal FC across both chromophores, alongside network-specific FC increases and decreases. Within the depressive-state group, the DP_AQ-high subgroup consistently demonstrated increased interhemispheric frontoparietal FC across both chromophores, accompanied by localized intra-frontal FC reductions, compared with the DP_AQ-low subgroup.

**Conclusion:**

These findings suggest that autistic traits modulate task-evoked large-scale network organization within a clinically relevant depressive-state cohort and support the utility of fNIRS-based network analysis for characterizing neurobiological heterogeneity associated with depressive symptoms.

## 1 Introduction

Autism spectrum disorder (ASD) is a neurodevelopmental disorder characterized by persistent deficits in social communication and social interaction, and by restricted and repetitive patterns of behavior, interests, and activities (American Psychiatric Association, 2013). Its prevalence is estimated to be approximately 0.7% globally (Baxter et al., 2015; Talantseva et al., 2023). Approximately 6%–16% of individuals experiencing depressive symptoms have an ASD diagnosis (Takara and Kondo, 2014; Radtke et al., 2019) and approximately 45%–50% exceed the cutoff values on ASD-related measures such as the Social Responsiveness Scale (SRS) or the Autism-Spectrum Quotient (AQ) (Matsuo et al., 2015; Radtke et al., 2019); the probability that individuals experiencing depressive symptoms have an ASD diagnosis or ASD traits is higher than that observed in neurotypical controls or the general population. Comorbidity between depression and ASD has been reported to frequently occur, and it gives rise to significant clinical problems. Specifically, individuals experiencing depressive symptoms who also have ASD show reduced treatment responsiveness (El Baou et al., 2023), a higher likelihood of attempting more lethal methods when planning suicide (Takara and Kondo, 2014), and increased diagnostic difficulty due to symptom overlap (diagnostic overshadowing) (Gupta and Gupta, 2023).

To address these issues, objectively elucidating the neural substrates of comorbid cases using functional near-infrared spectroscopy (fNIRS) may be a meaningful approach. fNIRS is a non-invasive and safe neuroimaging technique that measures changes in oxyhemoglobin and deoxyhemoglobin (HbO_2_/HbR) in the superficial cerebral cortex by emitting near-infrared light through the scalp. Because the device is quiet, relatively portable, and easy to wear, fNIRS allows measurements without the strong physical constraints required for functional magnetic resonance imaging (fMRI) and can be performed while participants maintain natural postures such as sitting or standing (Pinti et al., 2020). Accordingly, fNIRS has been widely used in clinical settings and clinical research (Lai et al., 2017; Chen et al., 2020). For example, a study reported in 2014 suggested that applying fNIRS to detect reduced frontal and temporal activation as a biomarker for depression can be clinically useful for supporting differential diagnosis (Takizawa et al., 2014; Fukuda, 2015). In addition, in ASD classification, resting-state functional connectivity (FC) analysis using fNIRS has demonstrated a high accuracy of 94% (Li et al., 2023). With regard to treatment support, fNIRS has been used to detect increased right prefrontal activation following methylphenidate (MPH) administration in attention deficit hyperactivity disorder (ADHD) (Monden et al., 2012; Nagashima et al., 2014), as well as to detect activation of the prefrontal cortex in patients with major depressive disorder (MDD) following transcranial magnetic stimulation (TMS) (Huang et al., 2022). These findings indicate that fNIRS can be used to quantify treatment-related effects. Moreover, a study focusing on comorbid ADHD and ASD (Sutoko et al., 2019) used fNIRS to detect MPH-induced brain activation, suggesting that fNIRS-based elucidation of neural substrates may be applicable to clinical support for comorbid cases as well.

Currently, what is known about the neural substrates of ASD and depression is summarized as follows. In ASD, reduced long-range resting-state FC, particularly within frontoparietal networks (FPN), has been consistently reported (Anderson et al., 2011; Mohammad-Rezazadeh et al., 2016). In depression, decreased connectivity within the central executive network (CEN) at rest, as well as an imbalance between the CEN and the default mode network (DMN), has been suggested to be associated with impairments in executive functioning (Hamilton et al., 2015; Kaiser et al., 2015; Mulders et al., 2015). In studies examining task-evoked FC in ASD, reduced prefrontal connectivity has been reported during the Wisconsin Card Sorting Test (WCST) (Chan et al., 2022a) and during verbal fluency tasks (VFT) (Chan et al., 2022b), and reduced intra-right prefrontal connectivity has also been reported during the n-back task (Han et al., 2022). These findings consistently indicate reduced intra-frontal FC during tasks that require cognitive control in individuals with ASD. Similarly, task-based FC studies in depression have reported reduced intra-frontal FC during VFT performance (Dong et al., 2021). Thus, we see that both disorders show reduced intra-frontal FC during cognitively demanding tasks. In contrast, studies focusing on comorbid conditions remain limited. Existing research has reported aberrant FC within the left salience network (SN) and cerebellar networks at rest (Kaneko et al., 2023; Nakamura et al., 2023), as well as stepwise reductions in right ventrolateral prefrontal cortex (VLPFC) activation associated with comorbidity during VFT performance (Ohtani et al., 2021). However, to the best of our knowledge, no studies have directly examined task-evoked FC in individuals experiencing depressive symptoms who exhibit ASD traits. Nevertheless, examining task-evoked FC is crucial for elucidating the neural substrates underlying the comorbidity of ASD and depression. Both ASD and depression have been conceptualized not merely as disorders involving localized functional abnormalities, but as conditions characterized by large-scale network dysfunction (Just et al., 2004; Kaiser et al., 2015; Hull et al., 2017; Hong et al., 2019). Moreover, a study employing a VFT in individuals with ASD demonstrated group differences relative to neurotypical controls in FC rather than in task-evoked activation (Chan et al., 2022b), further underscoring the importance of investigating disease-related neural alterations at the network level. In addition, tasks—particularly the VFT—are standardized clinical assessments that can be administered in a short time (Machado et al., 2009; Henderson et al., 2023). Because VFT performance involves not only lexical retrieval and language processing but also executive demands such as maintaining task rules, inhibiting repetitions, and switching (Whiteside et al., 2016), the VFT has been widely used to evaluate cognitive functions and their neural substrates relevant to psychiatric disorders. Therefore, examining FC during the VFT is useful for elucidating the neural substrates of comorbidity.

In the present study, depressive disorders were not restricted to MDD but were conceptualized to include subthreshold depression. Subthreshold depression has been regarded as a prodromal or early stage of MDD and has been associated with an increased risk of subsequent onset of major depression (Lee et al., 2019; Hao et al., 2023). From a clinical perspective, individuals with subthreshold depressive symptoms frequently present to psychiatric services and constitute an important target population for early detection and intervention (Li et al., 2024; National Institute for Health and Care Excellence (NICE), 2022). Consistent with this view, previous fNIRS studies have reported altered prefrontal activation and FC during a VFT even in individuals with subthreshold depressive symptoms, indicating that neurofunctional alterations can be detected before the onset of full-syndromal MDD (Da et al., 2024).

In parallel, autistic characteristics were assessed using the AQ rather than relying on a categorical ASD diagnosis. Autistic traits are continuously distributed in the general population, and dimensional approaches using AQ scores have been increasingly adopted to capture inter-individual variability in social communication, cognitive flexibility, and executive functioning (Kamio et al., 2013; Wittkopf et al., 2023). Importantly, individuals with elevated autistic traits may experience clinically significant difficulties even in the absence of a formal ASD diagnosis, particularly in the context of comorbid affective symptoms (Takara and Kondo, 2014; Radtke et al., 2019).

Based on this framework, the present study aimed to clarify how FC during VFT performance differs as a function of autistic traits in individuals experiencing depressive symptoms. Specifically, we examined whether individuals experiencing depressive symptoms with high AQ scores show altered task-evoked FC compared with those with lower AQ scores. With regard to task-related language networks, previous studies have suggested that left fronto-temporal connectivity is primarily associated with core language processing and verbal fluency performance, whereas right temporal regions play a critical role in social cognition and socio-emotional processing. On the basis of these findings, we hypothesized that, during VFT performance, individuals experiencing depressive symptoms with high AQ scores would not show marked differences in left fronto-temporal FC relative to those with low AQ scores but would exhibit reduced FC between the left prefrontal and right temporal regions. This pattern was expected to reflect additional network-level vulnerability associated with autistic traits in the context of depression. Accordingly, the present study used fNIRS to test this hypothesis by comparing task-evoked FC during VFT performance among neurotypical controls, individuals experiencing depressive symptoms with low AQ scores, and individuals experiencing depressive symptoms with high AQ scores.

## 2 Materials and methods

### 2.1 Ethical considerations

This study was approved by the Ethics Committee of Nippon Medical School Chiba Hokusoh Hospital (Approval Number: H27-457). All participants received a thorough explanation of the study objectives and procedures, and written informed consent was obtained prior to participation.

### 2.2 Participants

Fifty neurotypical controls (40 males, 10 females; mean age 31.5 ± 7.5 years) and 43 patients presenting with depressive symptoms (22 males, 21 females; mean age 35.6 ± 12.9 years) participated in this study. All participants in the neurotypical control group were selected from a database of 50 neurotypical individuals registered at Nippon Medical School Chiba Hokusoh Hospital.

All participants in the depression (DP) group were recruited from the outpatient psychiatric department at Nippon Medical School Chiba Hokusoh Hospital. Although one participant was a native Chinese speaker, this individual had lived in Japan for 15 years and had been clinically assessed to have sufficient Japanese proficiency to understand the task instructions and perform the phonemic verbal fluency task. No participants were excluded solely on the basis of native-language status.

Participants in the patient group underwent fNIRS assessment during a VFT as part of a clinical evaluation. The patient cohort was defined as a clinically relevant depressive-state cohort rather than a single diagnostic category. The diagnostic composition of the patient cohort was heterogeneous and included individuals with major depressive disorder or recurrent depressive disorder, bipolar affective disorder in depressive or mixed states, and other depressive conditions mainly characterized by dysthymic symptoms. Several participants also presented with comorbid neurodevelopmental or anxiety-related conditions (e.g., autistic traits, ADHD, and anxiety disorders), reflecting the heterogeneity commonly encountered in psychiatric outpatient populations. The diagnostic composition of the cohort is summarized in Supplementary Table S1.

Autistic traits were assessed using the AQ. Patients were divided into two groups according to AQ score, resulting in an AQ-high group (*n* = 20) and an AQ-low group (*n* = 23). Age and sex distributions were comparable between the two groups. The aim of the present study was to examine FC associated with autistic traits within a clinically relevant depressive-state cohort rather than within a single diagnostic category.

### 2.3 Diagnosis and AQ-based grouping

Clinical diagnoses were made by a board-certified psychiatrist based on clinical interviews and medical record review based on ICD-10 criteria, and diagnostic information was also interpreted with reference to DSM-5 terminology for clarity (American Psychiatric Association, 2013).

All patients were experiencing clinically significant depressive symptoms at the time of fNIRS measurement and had a Hamilton Depression Rating Scale (HAM-D) score of 8 or higher (Hamilton, 1960). The Patient Health Questionnaire-9 (PHQ-9) and the Quick Inventory of Depressive Symptomatology (QIDS) were also administered prior to measurement (Kroenke et al., 2001; Rush et al., 2003). The DP group was classified into two subgroups based on AQ scores: (1) the DP_AQ-high group (AQ score ≥ 33; 10 males and 10 females; mean age 36.7 ± 14.3 years) and (2) the DP_AQ-low group (AQ score < 33; 12 males and 11 females; mean age 34.7 ± 11.9 years). For the Japanese version of the AQ, a cutoff score of 33 has been reported as valid (Wakabayashi et al., 2006).

The cutoff-based grouping was used to define clinically interpretable strata of autistic traits within the depressive-state cohort. This grouping was not intended to classify participants as having or not having ASD, but rather to provide an exploratory stratification for examining task-evoked FC differences associated with relatively high versus low autistic traits. We acknowledge that AQ is inherently dimensional, and the cutoff-based grouping should therefore be interpreted as a pragmatic analytic approach rather than a categorical diagnostic distinction.

### 2.4 Verbal fluency task

**Figure 1:**
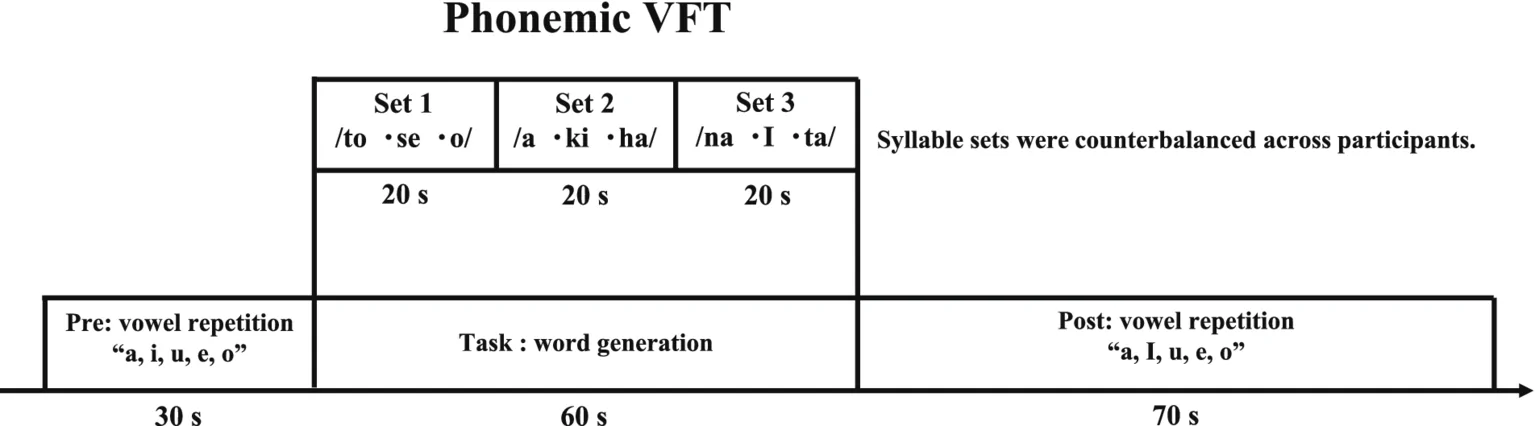
Timeline of the phonemic verbal fluency task (procedure follows Akiyama et al., 2018). Participants repeated Japanese vowels during baseline segments and generated nouns based on syllable cues presented during the task segment.

Participants performed a phonemic VFT (Figure 1). Following previous research (Akiyama et al., 2018), the task consisted of the following three segments:

- Pre-baseline segment: 30 s.
- Task segment: 60 s.
- Post-baseline segment: 70 s.

During the pre- and post-baseline segments, participants were instructed to repeatedly vocalize the Japanese vowels “a, i, u, e, o” in sequence. In the task segment, participants were asked to generate as many nouns as possible starting with a specified phoneme, avoiding repetition and proper nouns. Three phoneme sets were used (/to·se·o/, /a·ki·ha/, /na·i·ta/), switched every 20 s, and counterbalanced across subjects. The number of utterances during the task was recorded as an indicator of task performance.

### 2.5 fNIRS measurement and channel localization

**Figure 2:**
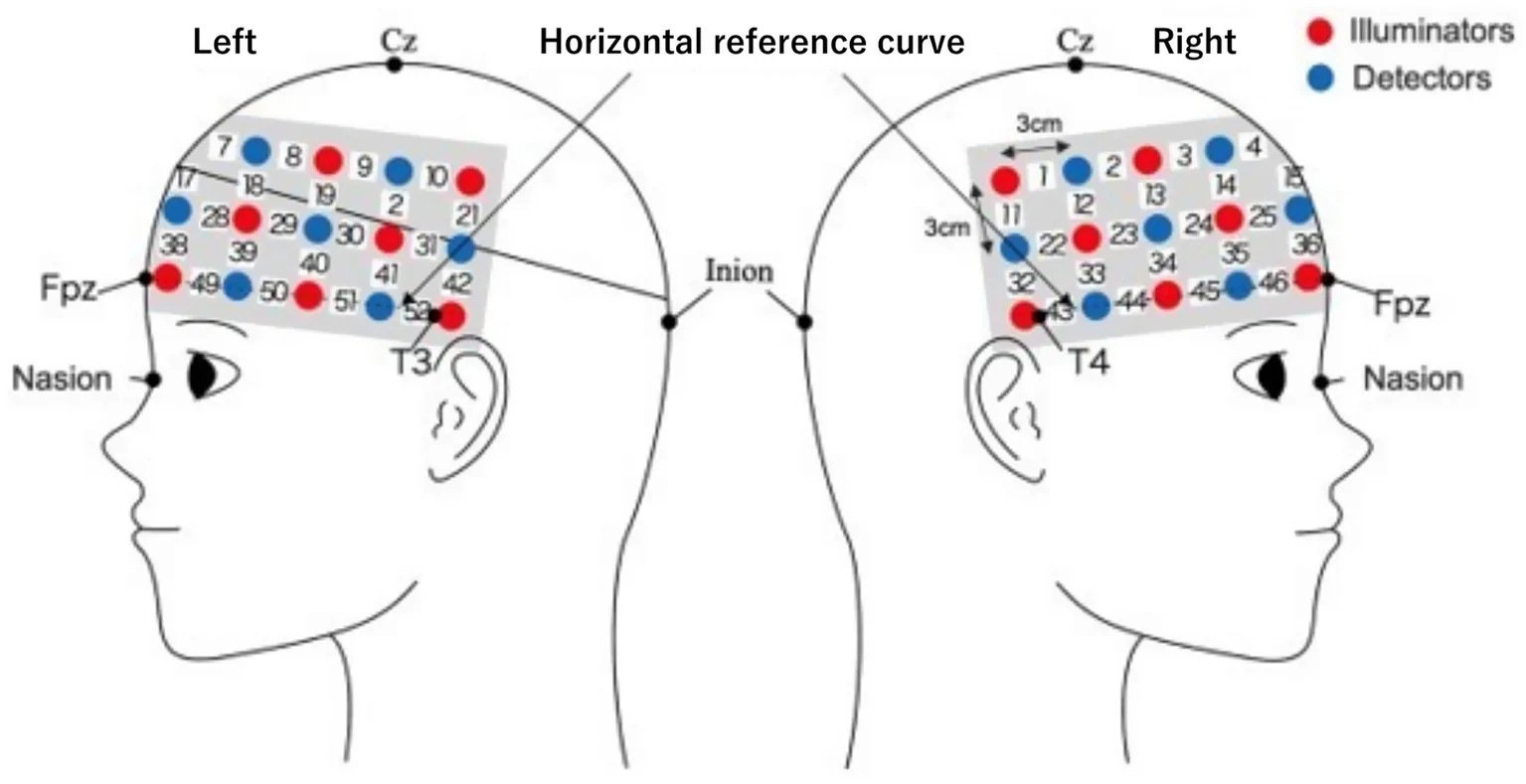
Probe configuration and channel layout. The optode array consisted of 17 emitters (red) and 16 detectors (blue) arranged in a 3 × 11 matrix with a 3-cm emitter–detector distance. Numbers indicate measurement channels (CH1–CH52); major scalp landmarks are shown for orientation.

**Figure 3:**
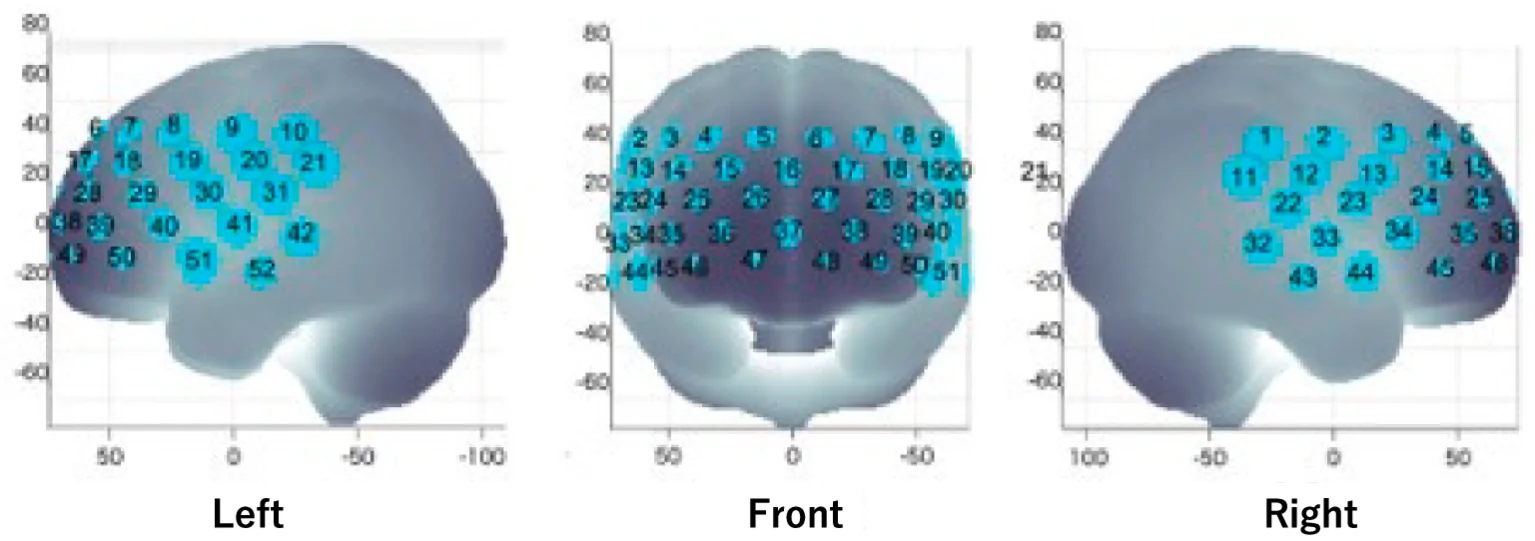
Projection of fNIRS channels onto MNI space. Cortical locations of the measurement channels were estimated and projected onto Montreal Neurological Institute (MNI) space using the virtual registration and anchor registration methods (Tsuzuki et al., 2007, 2012). Cyan markers denote the projected positions of CH1–CH52 shown from left, frontal, and right views.

A 52-channel fNIRS device (ETG-4000, Hitachi Medical Corporation, Tokyo, Japan) was used to measure the relative changes in HbO_2_ and HbR. Light source wavelengths were 695 nm and 830 nm, and the modified Beer–Lambert law was applied. The sampling frequency was 10 Hz (0.1-s intervals). An optode array consisting of 17 light emitters and 16 light detectors was arranged in a 3 × 11 grid configuration, with an emitter-detector distance of 3 cm (Figure 2). The cortical locations of the measurement channels were projected onto MNI space using virtual registration and anchor registration methods (Tsuzuki et al., 2007, 2012) (Figure 3) with the LPBA40 atlas referenced for identifying corresponding anatomical regions. The full channel-wise correspondence between fNIRS channels, MNI coordinates, and LPBA40 anatomical labels is provided in Supplementary Data Sheet 1. For clarity in this paper, the superior, middle, and inferior frontal gyri as well as the precentral gyrus are referred to as the frontal lobe; the superior, middle, and inferior temporal gyri as the temporal lobe; and the supramarginal gyrus as the inferior parietal lobule (IPL).

### 2.6 Data preprocessing

Raw fNIRS intensity signals were first converted to optical density using a baseline length of 5 samples and then transformed into HbO₂ and HbR concentration changes using the modified Beer–Lambert law. Channel quality was evaluated before functional connectivity analysis. Specifically, scalp coupling and signal quality were assessed using the scalp coupling index (SCI), peak spectral power (PSP), and signal-to-noise ratio (SNR). SCI and PSP were calculated after applying a 0.8–1.5 Hz first-order Butterworth band-pass filter to the two-wavelength optical density signals. SCI was calculated as the zero-lag cross-correlation between the two wavelength signals within the cardiac frequency band, and PSP was calculated as the peak spectral power of the cross-correlation spectrum. Channels that did not meet the predefined SCI, PSP, or SNR criteria were excluded from subsequent analyses; specifically, these were channels with SCI < 0.25, PSP < 0.10, or SNR < 20 dB.

Motion artifacts were detected using a moving-standard-deviation-based procedure and Homer-type motion detection indices. The main parameters used for motion detection, including the window length, Tmotion, masking interval, adaptive standard deviation threshold, and amplitude threshold, were specified *a priori*. Specifically, motion detection was performed using a moving standard deviation (MSD) window of 0.5 s, Tmotion of 0.5 s, a masking interval of 1.0 s, a low fraction of 1/3, and a mask mode of 3, which combined adaptive standard deviation and amplitude-based detection (AMP). The motion detection thresholds were set as follows: MSD = 0.03, standard deviation = 4.0, AMP = 0.5, and adaptive standard deviation = 4.0. Detected motion-contaminated segments were corrected using spline interpolation and wavelet-based correction. The spline parameter and wavelet threshold were set according to the preprocessing protocol and are reported to ensure reproducibility; specifically, the spline interpolation parameters were *p* = 0.99, short shift = 0.3 s, and long shift = 3.0 s, and the wavelet-based correction parameters were wavelet = db2, interquartile range threshold = 1.0, lowestScale = 4, maxLevel = 6.

To reduce the influence of shared global components that may reflect superficial blood flow and systemic physiological fluctuations, principal component analysis (PCA)/coefficient of spatial uniformity (CSU)-based global component correction was applied to the HbO₂ and HbR time series prior to FC calculation. In the PCA/CSU-based correction, Step 1, Step 2, and Step 3 of the CSU removal procedure were applied separately to the HbO₂ and HbR signals, and components exceeding the predefined CSU threshold were removed before FC calculation. Finally, a band-pass filter of 0.009–0.1 Hz was applied to the preprocessed HbO₂ and HbR signals using a first-order Butterworth IIR (infinite impulse response) filter to retain low-frequency hemodynamic fluctuations used for task-evoked fNIRS FC analysis (Sasai et al., 2011).

### 2.7 FC calculation

FC was calculated from the 60-s task segment using preprocessed HbO_2_ and HbR time series. FC strength was defined as the temporal correlation of hemodynamic responses between each channel pair. Pearson’s correlation coefficient (*r*) was calculated at zero lag for each unique channel pair. The resulting correlation coefficients were converted to z values using Fisher’s *r*-to-*z* transformation (Fisher, 1915) to improve normality and were used for statistical analyses.

### 2.8 Statistical analysis: demographic and clinical variables

A two-sample t-test was performed to compare age, age at onset, duration of illness, depression severity (HAM-D, PHQ-9, QIDS), anxiety severity (GAD-7), medication dosage, and AQ scores (total and subscale) between the DP_AQ-high and DP_AQ-low groups. Homogeneity of variance was confirmed using Levene’s test. For medication variables, antidepressant, anxiolytic, and antipsychotic dosages were converted to imipramine-, diazepam-, and chlorpromazine-equivalent doses (mg/day), respectively, to allow comparison across different agents.

### 2.9 Statistical analysis: task performance and FC

One-way randomized-groups analysis of variance (ANOVA) was performed with the VFT word counts and z scores of each channel pair as the dependent variables and group (Control, DP_AQ-low, DP_AQ-high) as the independent variable. For FC analyses, the statistical model differed in contrast. For Contrast 1, comparing the Control group with the depressive-state cohort, one-way ANOVA was performed for each channel pair using Fisher z-transformed FC values as the dependent variable and group as the independent variable. For Contrast 2, comparing DP_AQ-high and DP_AQ-low groups, analysis of covariance (ANCOVA) was performed for each channel pair using Fisher z-transformed FC values as the dependent variable, AQ-based group as the main effect, and anxiolytic and antidepressant dosages as covariates. Anxiolytic dosage was entered as diazepam-equivalent dose, and antidepressant dosage was entered as imipramine-equivalent dose. Because the Control participants were not taking psychotropic medications, coding their medication dosage as zero would have made medication use highly collinear with group status. Therefore, medication-adjusted ANCOVA was applied only to the within-patient comparison between the DP_AQ-high and DP_AQ-low groups. Subsequently, planned comparisons were based on two predefined contrasts:

- Contrast 1: control group vs. DP group.
- Contrast 2: DP_AQ-high group vs. DP_AQ-low group.

**Figure 4:**
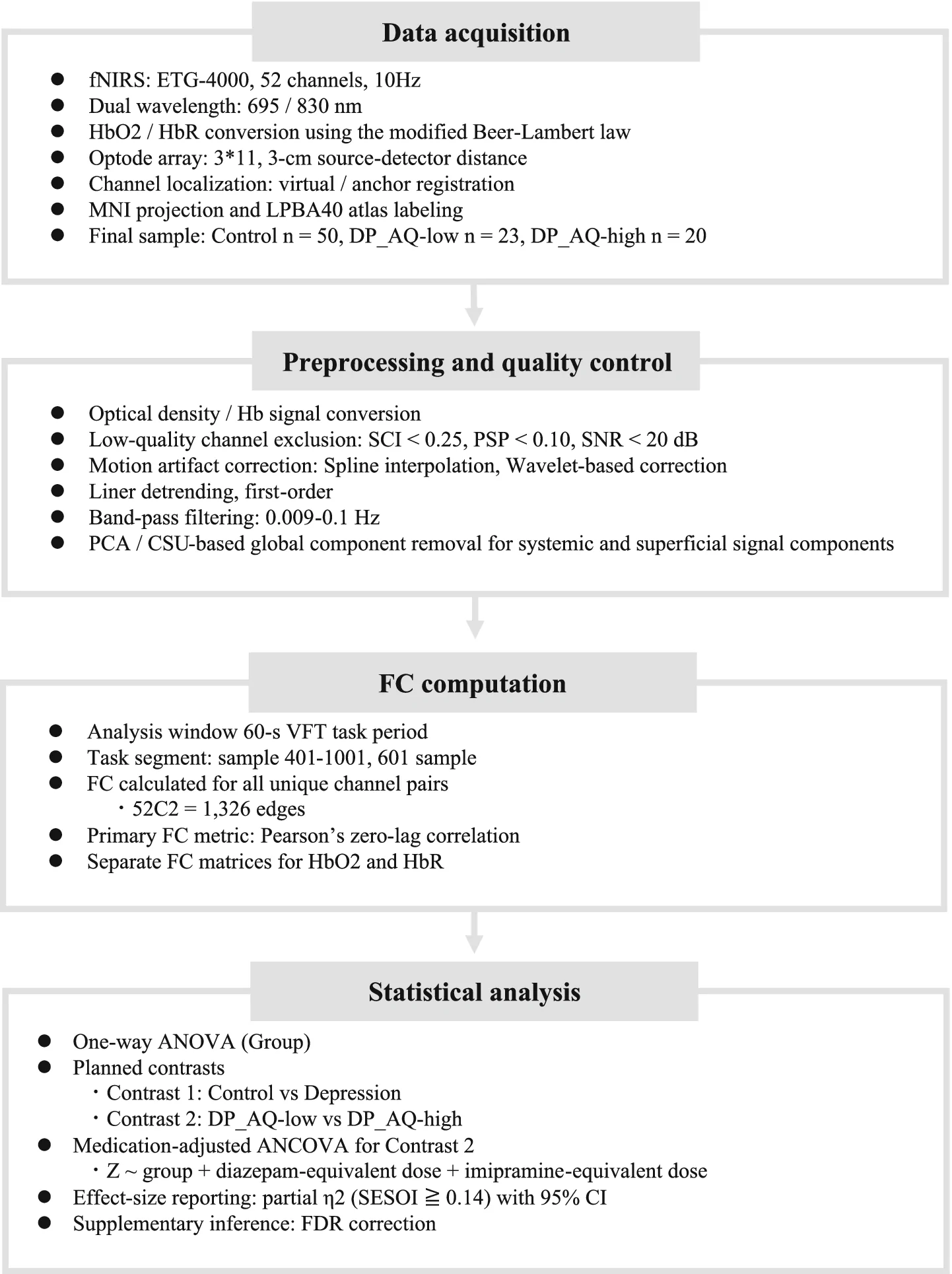
Analysis pipeline: schematic overview of the workflow from fNIRS data acquisition and preprocessing to functional connectivity (FC) computation (HbO_2_ and HbR) and statistical analyses.

This study interpreted between-group differences primarily on the basis of effect sizes (partial *η*^2^), rather than relying on a dichotomous significant/non-significant decision based on *p* values. Dichotomizing results by p values has been argued to distort interpretation (Wasserstein and Lazar, 2016). Therefore, from the perspectives of interpretability and the role of effect sizes in cumulative science (e.g., meta-analysis), we judged an effect-size-based interpretation to be appropriate (Cumming, 2014). However, for visualization and interpretation of between-group patterns, we predefined a smallest effect size of interest (SESOI) (Lakens et al., 2018). Specifically, we selected partial *η*^2^ = 0.14—often used as a benchmark for a “large” effect in ANOVA (Cohen, 1988)—as the SESOI and reported 95% confidence intervals for each effect size. It should be noted, however, that this threshold-based classification inherently relies on point estimates; thus, the precision and reliability of the designated effects must be evaluated with caution by inspecting their corresponding confidence intervals. For transparency, raw *p*-values and Benjamini–Hochberg false-discover-rate (BH-FDR)-adjusted *q* values were also computed across all unique channel pairs within each contrast and hemoglobin signal and are provided in the corresponding Results tables together with the effect sizes and their 95% confidence intervals. An overview of the analysis pipeline is provided in Figure 4. All statistical analyses were performed using MATLAB 2022b/2025b and SPSS Graphs and significance networks were generated and visualized using MATLAB-based scripts.

## 3 Results

### 3.1 Demographic and clinical characteristics

Demographic and clinical characteristics for each group are shown in Table 1. No significant differences were observed between the AQ-high and AQ-low groups in age, age at onset, duration of illness, depression severity (HAM-D, PHQ-9, QIDS), anxiety severity (GAD-7), dosage of any of the three psychotropic medications (antidepressants, anxiolytics, or antipsychotics), or the AQ attention to detail subscale score. In contrast, both the total AQ score and the other four subscale scores (social skills, attention switching, communication, and imagination) were significantly higher in the AQ-high group. These results confirm that grouping based on AQ scores appropriately reflects individual differences in ASD characteristics.

**Table 1:** Demographic and neuropsychological characteristics of DP_AQ-low and DP_AQ-high groups.

Variable	DP_AQ-high	DP_AQ-low	*p*-value
Number of participants	20	23	
Sex (male/female)	10/10	12/11	N.S.
Age (years)	36.7 ± 14.3	34.7 ± 14.3	N.S.
Age at onset (years)	28.6 ± 14.5	26.9 ± 10.4	N.S.
Duration of illness (years)	7.7 ± 7.6	7.8 ± 9.6	N.S.
HAM-D (first visit)	17.2 ± 4.5	14.6 ± 4.6	N.S.
PHQ-9 (pre-experiment)	18.5 ± 6.1	15.7 ± 4.8	N.S.
QIDS	17.3 ± 4.9	15.3 ± 4.6	N.S.
GAD-7	11.8 ± 5.1	11.3 ± 5.7	N.S.
AQ total	37.4 ± 2.7**	23.3 ± 5.3**	<0.001
AQ social skills	8.5 ± 0.9**	4.9 ± 3.0**	<0.001
AQ attention switching	8.0 ± 1.1**	5.4 ± 1.5**	<0.001
AQ attention to detail	5.4 ± 2.1	5.0 ± 2.2	N.S.
AQ communication	8.3 ± 1.2**	4.1 ± 2.0**	<0.001
AQ imagination	7.3 ± 1.5**	3.8 ± 2.0**	<0.001
Antidepressant dosage	80.6 ± 110.2	74.2 ± 102.8	N.S.
Anxiolytic dosage	10.6 ± 14.2	7.2 ± 7.3	N.S.
Antipsychotic dosage	76.8 ± 142.4	41.3 ± 115.1	N.S.

Values are presented as mean ± SD unless otherwise indicated. Group comparisons were performed using independent-samples t-tests (two-tailed), except for sex ratios, which were compared using the chi-square test. Antidepressant, anxiolytic, and antipsychotic dosages were calculated as imipramine-, diazepam-, and chlorpromazine-equivalent doses (mg/day), respectively. DP, depression group, AQ, the autism-spectrum quotient, HAM-D, Hamilton depression rating scale; PHQ-9, the patient health questionnaire-9, QIDS, quick inventory of depressive symptomatology.

Values are presented as mean ± SD unless otherwise indicated. Group comparisons were performed using independent-samples t-tests (two-tailed), except for sex ratios, which were compared using the chi-square test. Antidepressant, anxiolytic, and antipsychotic dosages were calculated as imipramine-, diazepam-, and chlorpromazine-equivalent doses (mg/day), respectively. Abbreviations: DP: depression group, AQ: the Autism-Spectrum Quotient, HAM-D: Hamilton Depression Rating Scale; PHQ-9: the Patient Health Questionnaire-9, QIDS: Quick Inventory of Depressive Symptomatology.

### 3.2 Task performance

The total number of words produced during the VFT was compared using the pre-planned contrasts described above. Contrast 1 (Control vs. DP) did not yield a significant difference in the number of words produced (DP_AQ-low: 13.61 ± 4.87; DP_AQ-high: 13.95 ± 5.20; Control: 15.72 ± 4.84), *F*(1, 90) = 3.58, *p* = 0.0617, *η*_p_^2^ = 0.0383, 95% CI [0.000, 0.142]. Contrast 2 (DP_AQ-low vs. DP_AQ-high) also did not yield a significant difference (DP_AQ-low: 13.61 ± 4.87; DP_AQ-high: 13.95 ± 5.20), *F*(1, 90) = 0.0514, *p* = 0.821, *η*_p_^2^ = 0.00057, 95% CI [0.000, 0.043].

### 3.3 FC analysis

During the VFT, zero-lag Pearson correlations were computed for the HbO₂ and HbR signals within the 60-s task period. The resulting correlation coefficients were Fisher z-transformed, and an ANCOVA with *a priori* planned contrasts was conducted for each channel pair, including the equivalent dosages of two medications (diazepam and imipramine) as covariates.

#### 3.3.1 Contrast 1: FC differences between control and DP

**Figure 5:**
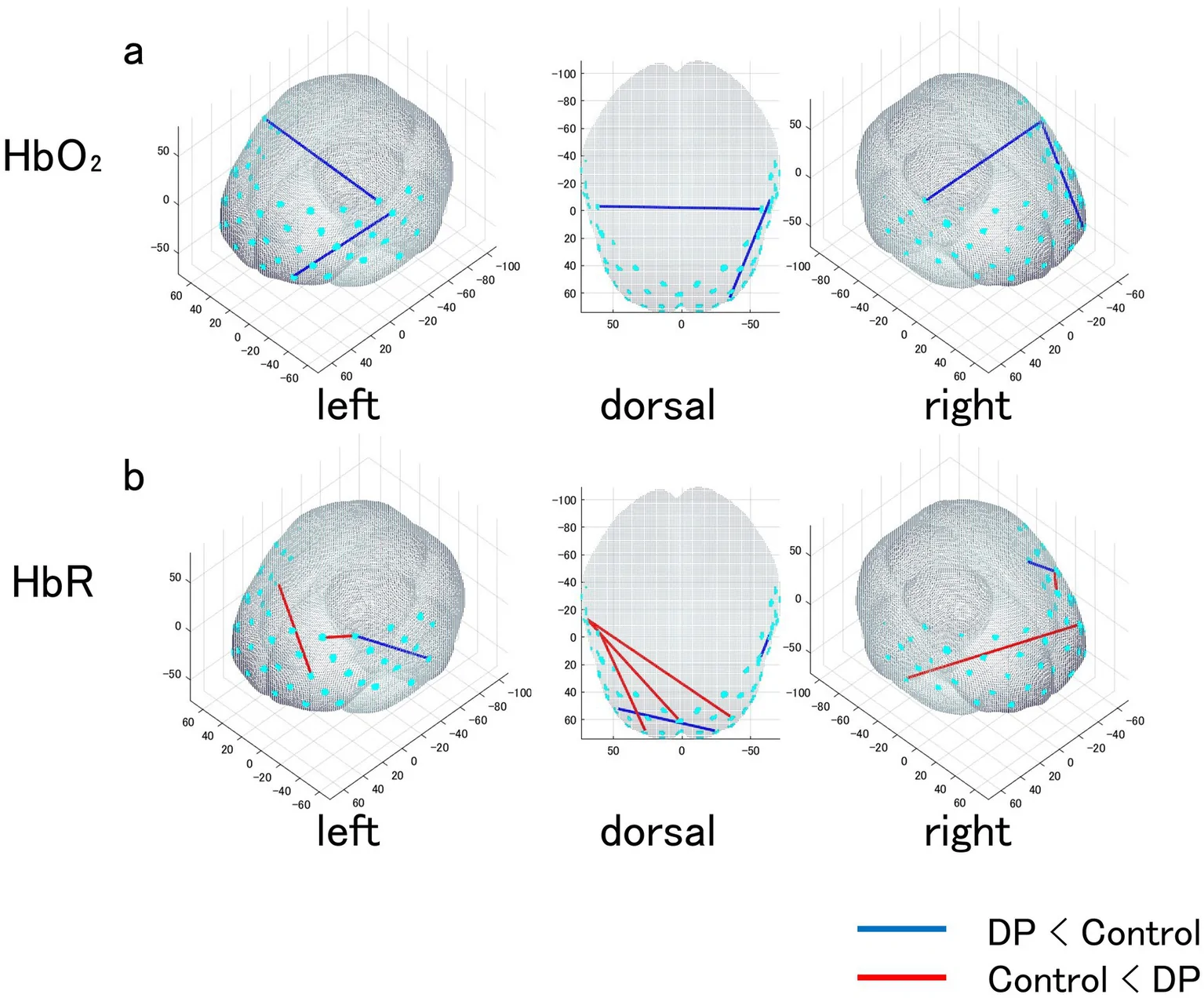
Channel-pair functional connectivity differences with large effect sizes (partial *η*^2^ ≥ 0.14) for Contrast 1 (Control vs. DP). This exploratory edgewise visualization was based on SESOI thresholding. Connections exceeding the smallest effect size of interest (SESOI; partial *η*^2^ ≥ 0.14) are visualized on the optode–channel layout for HbO_2_ **(a)** and HbR **(b)**. Blue lines indicate channel pairs showing higher connectivity in the Control group than in the DP group (Control > DP), whereas red lines indicate channel pairs showing higher connectivity in the DP group than in the Control group (DP > Control).

For Contrast 1, channel pairs showing FC differences that exceeded 0.14, defined as the SESOI, are presented in Figure 5 and Tables 2, 3. No edges survived BH-FDR correction (minimum q = 0.293). Given that this exploratory classification relies on point estimates, the corresponding confidence intervals (Tables 2, 3) should be considered.

**Table 2:** Contrast 1 (control vs. DP): task-state HbO_2_ functional connectivity edges exhibiting large effects.

HbO_2_					
Channel pairs	Brain regions	*η*_p_^2^	95% CI	*p*-value	*q*-value
Control > DP					
ch2-ch9	R PoCG - L PreCG	0.169	[0.026, 0.350]	0.002	0.350
ch20-ch49	L PoCG - L MFG	0.143	[0.026, 0.298]	0.001	0.350

Channel pairs with partial *ηp*^2^ > 0.14 are listed. Values are reported as *ηp*^2^ [95% CI], *p*-values, and *q*-values (adjusted using the Benjamini–Hochberg false discovery rate procedure); brain-region labels reflect anatomical channel assignments. L/R, left/right; PoCG, postcentral gyrus; PreCG, precentral gyrus; MFG, middle frontal gyrus.

**Table 3:** Contrast 1 (control vs. DP): task-state HbR functional connectivity edges exhibiting large effects.

HbR					
Channel pairs	Brain regions	*η*_p_^2^	95% CI	*p*-value	*q*-value
DP > control					
ch2-ch36	R PoCG–R MFG	0.146	[0.021, 0.314]	0.002	0.399
ch16-ch43	R SFG–R MTG	0.149	[0.026, 0.312]	0.001	0.399
ch28-ch43	L MFG–R MTG	0.161	[0.039, 0.313]	< 0.001	0.293
Control > DP					
ch35-ch38	R IFG–L MFG	0.146	[0.032, 0.291]	< 0.001	0.293
ch41-ch51	L STG–L STG	0.144	[0.024, 0.303]	0.001	0.399

Same analysis and reporting conventions as Table 2. L/R, left/right; PoCG, postcentral gyrus; MFG, middle frontal gyrus; SFG, superior frontal gyrus; MTG, middle temporal gyrus; IFG, inferior frontal gyrus; STG, superior temporal gyrus.

##### 3.3.1.1 HbO_2_

The between-group comparison showed that, for HbO₂, the DP group showed a trend toward reduced FC relative to the Control group between the left and right frontal regions, as well as between the left frontal and left parietal regions.

##### 3.3.1.2 HbR

For HbR, the DP group showed a trend toward increased FC relative to the Control group between the right frontal and right parietal regions, between the right frontal and right temporal regions, and between the left frontal and right temporal regions. Conversely, the DP group showed a trend toward reduced FC between the left and right frontal regions and within the left temporal region.

##### 3.3.1.3 HbO_2_–HbR convergence and divergence

**Figure 6:**
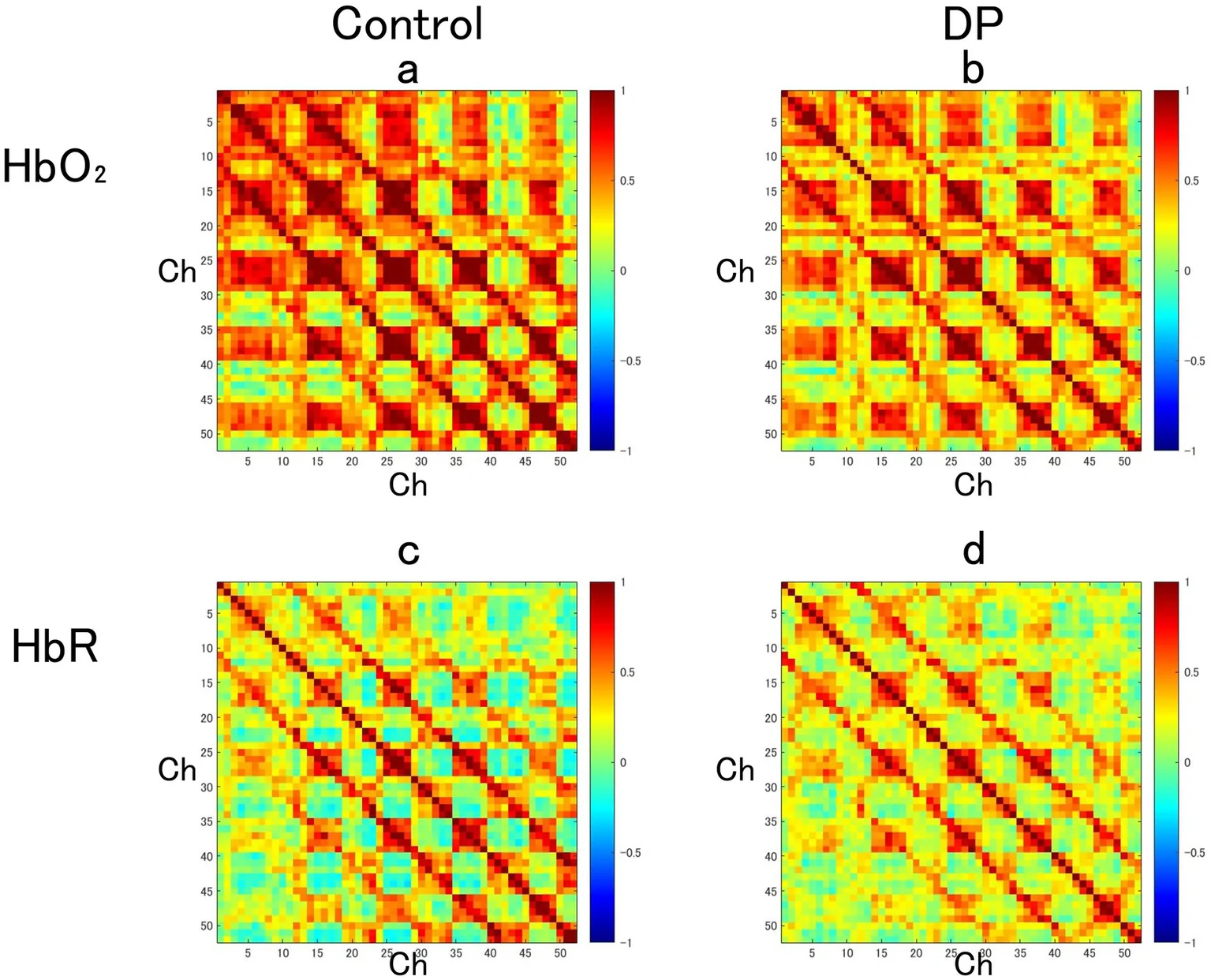
Group-averaged functional connectivity matrices during the verbal fluency task (VFT; Control and DP groups). Matrices display Pearson’s correlation coefficients. Panels show **(a)** Control, HbO_2_; **(b)** DP, HbO_2_; **(c)** Control, HbR; and **(d)** DP, HbR. The color scale indicates the strength of the correlation (*r*).

For both HbO₂ and HbR, the DP group consistently showed a trend toward reduced FC, relative to the Control group, between the left and right frontal regions. Respective divergences were observed in other connections: a reduction in FC was found between the left frontal and left parietal regions in HbO₂, and within the left temporal region in HbR, while several fronto-temporal and fronto-parietal connectivity increases were uniquely observed in the HbR signal. The FC maps for each group are shown in Figure 6.

#### 3.3.2 Contrast 2: FC differences between DP_AQ-low and DP_AQ-high

**Figure 7:**
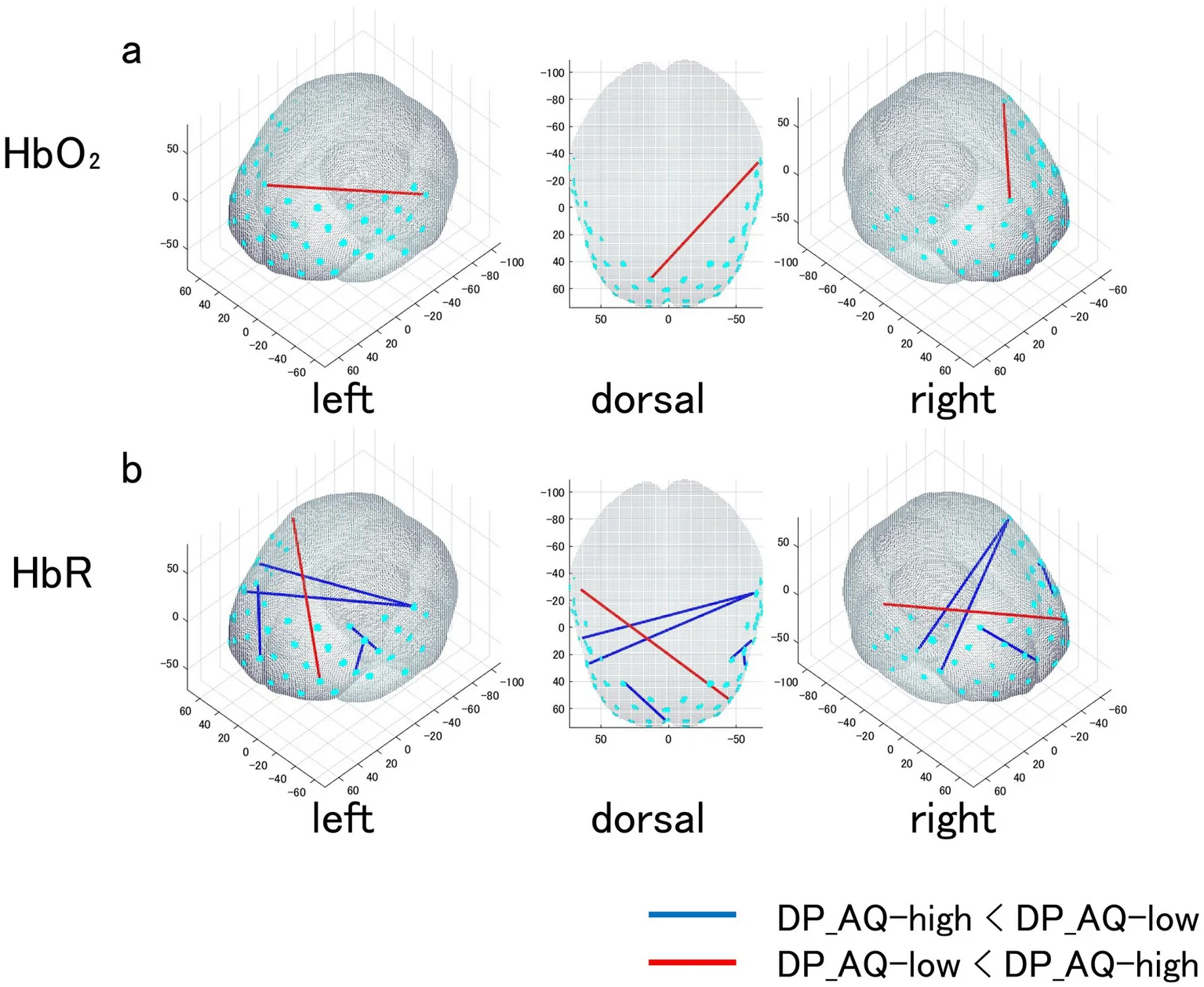
Channel-pair functional connectivity differences with large effect sizes (partial *η*^2^ ≥ 0.14) for Contrast 2 (DP_AQ-low vs. DP_AQ-high). This exploratory edgewise visualization was based on SESOI thresholding. Connections exceeding the smallest effect size of interest (SESOI; partial *η*^2^ ≥ 0.14) are visualized on the optode–channel layout for HbO **(a)** and HbR **(b)**. Blue lines indicate channel pairs showing higher connectivity in the DP_AQ-low group than in the DP_AQ-high group (DP_AQ-low > DP_AQ-high), whereas red lines indicate channel pairs showing higher connectivity in the DP_AQ-high group than in the DP_AQ-low group (DP_AQ-high > DP_AQ-low).

Channel pairs showing FC differences that exceeded 0.14, defined as the SESOI, in Contrast 2 are presented in Figure 7 and Tables 4, 5. Similar to Contrast 1, no edges survived BH-FDR correction (Tables 4, 5), and these findings should be regarded as exploratory.

**Table 4:** Contrast 2 (DP_AQ-low vs. DP_AQ-high): task-state HbO_2_ functional connectivity edges exhibiting large effects.

HbO_2_					
Channel pairs	Brain regions	*η*_p_^2^	95% CI	*p*-value	*q*-value
DP_AQ-high > DP_AQ-low					
ch5-ch21	R SFG–L SMG	0.143	[0.026, 0.297]	0.001	0.798

Same analysis and reporting conventions as Table 2. L/R, left/right; SFG, superior frontal gyrus; SMG, supramarginal gyrus.

**Table 5:** Contrast 2 (DP_AQ-low vs. DP_AQ-high): task-state HbR functional connectivity edges exhibiting large effects.

HbR					
Channel pairs	Brain regions	*η*_p_^2^	95% CI	*p*-value	*q*-value
DP_AQ-high > DP_AQ-low					
ch1-ch39	R SMG–L IFG	0.149	[0.026, 0.310]	0.001	0.419
DP_AQ-low > DP_AQ-high					
ch4-ch37	R MFG–R SFG	0.171	[0.033, 0.342]	0.001	0.408
ch8-ch30	L MFG–L PreCG	0.196	[0.031, 0.393]	0.002	0.453
ch10-ch23	L SMG–R PreCG	0.173	[0.035, 0.342]	0.001	0.408
ch10-ch34	L SMG–R IFG	0.142	[0.023, 0.302]	0.002	0.441
ch19-ch40	L MFG–L IFG	0.163	[0.036, 0.320]	<0.001	0.408

Same analysis and reporting conventions as Table 2. L/R, left/right; SMG, supramarginal gyrus; IFG, inferior frontal gyrus; MFG, middle frontal gyrus; SFG, superior frontal gyrus; PreCG, precentral gyrus.

##### 3.3.2.1 HbO_2_

For HbO₂, the group with autistic traits (DP_AQ-high) showed a trend toward increased FC between the right frontal and left parietal regions compared with the group without autistic traits (DP_AQ-low).

##### 3.3.2.2 HbR

For HbR, the group with autistic traits (DP_AQ-high) showed a trend toward increased FC between the left frontal and right parietal regions compared with the DP_AQ-low group. Conversely, the DP_AQ-high group exhibited reduced FC within the right frontal region, within the left frontal region, and between the left parietal and right frontal regions.

##### 3.3.2.3 HbO_2_–HbR convergence and divergence

**Figure 8:**
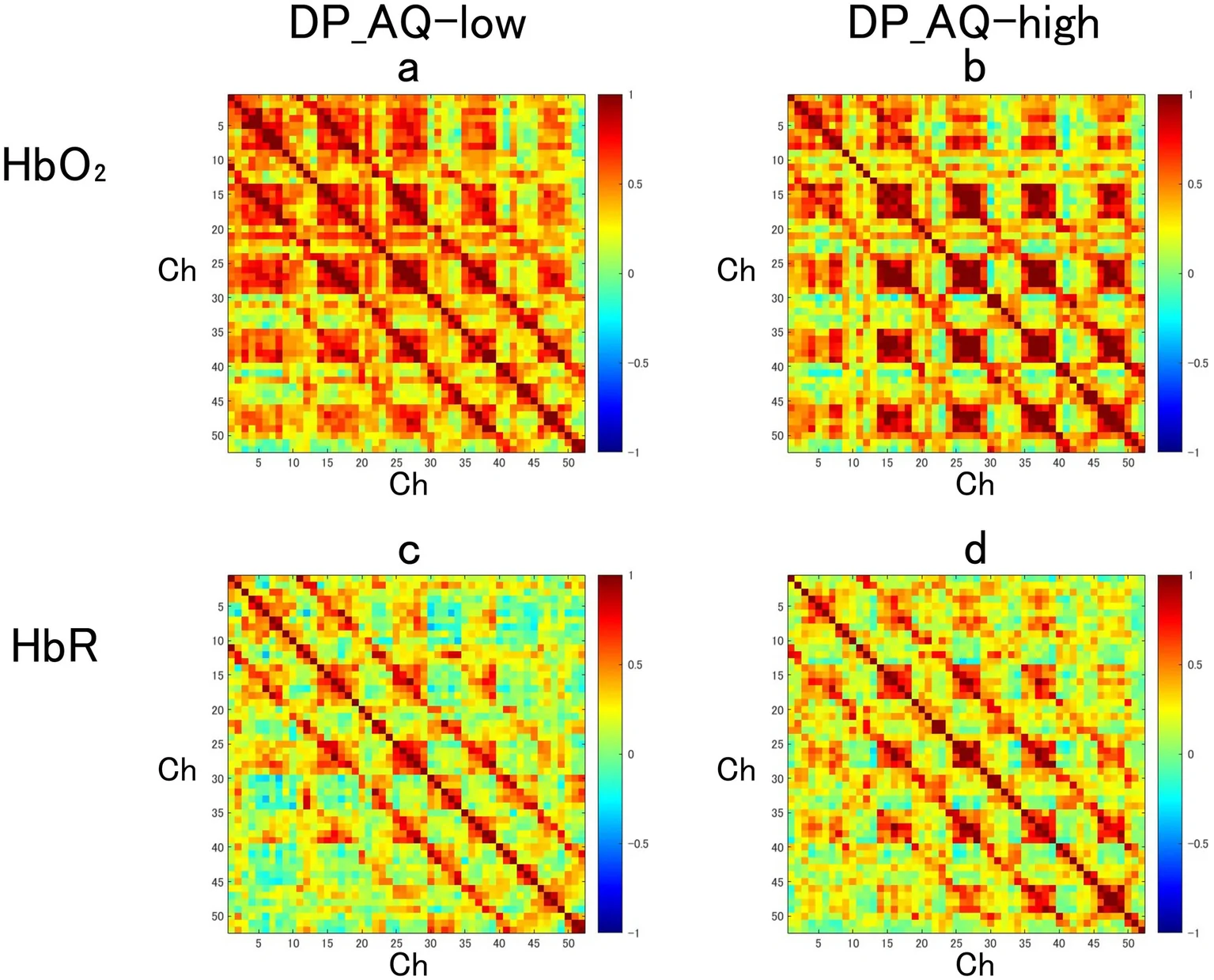
Group-averaged functional connectivity matrices during the verbal fluency task (VFT; DP_AQ-low and DP_AQ-high groups). Matrices display Pearson’s correlation coefficients. Panels show **(a)** DP_AQ-low, HbO_2_; **(b)** DP_AQ-high, HbO_2_; **(c)** DP_AQ-low, HbR; and **(d)** DP_AQ-high, HbR. The color scale indicates the strength of the correlation (*r*).

While no identical channel pairs showed common changes across both HbO₂ and HbR, both signals consistently revealed increased interhemispheric FC connecting the frontal and contralateral parietal regions in the DP_AQ-high group. In addition, HbR revealed more extensive reductions in FC within the bilateral frontal regions and between the left parietal and right frontal regions in the DP_AQ-high group. The FC maps for each group are shown in Figure 8.

## 4 Discussion

### 4.1 Summary of findings

The present study used fNIRS to investigate task-evoked FC during the performance of a VFT as a function of autistic traits in individuals experiencing depressive symptoms. Consistent with our hypothesis, individuals experiencing depressive symptoms showed suggestive patterns of altered FC within prefrontal and fronto-temporal networks relative to neurotypical controls, as reflected by decreased connectivity between the left and right frontal regions across both HbO₂ and HbR signals, alongside divergent changes in other prefrontal, fronto-temporal, and fronto-parietal pathways. Importantly, within the depression group, individuals with high AQ scores showed a reorganization of FC compared to those with low AQ scores. Across both chromophores, the AQ-high group consistently presented suggestive patterns of increased interhemispheric FC connecting the frontal and contralateral parietal regions, while reductions within the frontal regions and between the left parietal and right frontal regions suggested exploratory patterns, particularly in the HbR analysis. This complex pattern of hyper- and hypo-connectivity contradicts a simple linear deficit model, suggesting instead an alternative network routing during a VFT.

Furthermore, altered connectivity involving fronto-temporal pathways, including connections between prefrontal and temporal regions, were evident particularly in HbR analyses, suggesting altered integration between executive and auditory/phonological monitoring networks during the performance of a VFT. Together, these findings demonstrate profiles of task-evoked FC among neurotypical controls, individuals experiencing depressive symptom with low AQ scores, and individuals experiencing depressive symptom with high AQ scores. This pattern supports the notion that autistic traits modulate large-scale brain network function when concomitant with depressive symptoms, particularly within fronto-parietal and fronto-temporal systems engaged during cognitively demanding tasks.

### 4.2 Frontal cognitive executive network dysfunction in depression

The alterations in FC, specifically the decreased FC between the left and right frontal regions observed across both chromophores alongside reduced fronto-temporal connectivity in Contrast 1 (Control vs. DP), may potentially reflect altered cognitive control dynamics. In a study examining FC during the VFT in patients with MDD, widespread alterations, including decreases, in intra- and interhemispheric frontal FC were reported (Dong et al., 2021). Our finding of decreased interhemispheric frontal connectivity directly aligns with the decreases reported by Dong et al. (2021). Concurrently, the specific increases in fronto-temporal and fronto-parietal connectivity observed uniquely in the HbR signal may reflect a compensatory network reorganization to maintain equivalent task performance, given that the total number of words produced did not differ between the groups. In addition, the reduced FC between the left frontal and left parietal regions observed in the HbO₂ signal, as well as within the left temporal region in HbR, appears to align with evidence of disrupted large-scale network interactions, including fronto-temporal or central executive systems, in patients with MDD (Kaiser et al., 2015; Alexopoulos et al., 2012). The altered network connectivity reported in prior studies has been discussed in relation to MDD-associated impairments in executive function. In the present study, we have furthered that discussion to include comorbid ASD symptoms.

### 4.3 Impact of ASD traits on executive networks

In Contrast 2 (DP_AQ-low vs. DP_AQ-high), in the DP_AQ-high group, increased interhemispheric FC between the frontal and contralateral parietal regions consistently suggested an exploratory pattern across both chromophores (between the right frontal and left parietal regions in HbO₂; between the left frontal and right parietal regions in HbR). Concurrently, decreased FC within the frontal regions and between the left parietal and right frontal regions was observed in the HbR signal. These complex alterations in FC appear to align with findings in ASD that have often revealed abnormalities (both hyper- and hypo-connectivity) within the frontal lobe and between frontal and parietal regions involved in executive function and cognitive control, and they suggest a qualitative shift in network organization under executive-system load during VFT in individuals with depressive symptoms and comorbid ASD traits. For example, a task-based fNIRS study examining FC during a verbal fluency task in individuals with ASD reported altered or reduced intra-frontal FC, which was associated with difficulties in cognitive flexibility and executive processing (Chan et al., 2022b), which aligns with the intra-frontal reductions observed in our HbR data.

Furthermore, reviews synthesizing resting-state FC in ASD (Mohammad-Rezazadeh et al., 2016; Hull et al., 2017) have explicitly noted the coexistence of hyperconnectivity and hypoconnectivity while organizing evidence for the presence of network-level abnormalities, and have reported altered long-range FC, including alterations involving the CEN (Hull et al., 2017). Our findings of concurrent frontoparietal hyperconnectivity and intra-frontal hypoconnectivity directly mirror this coexistence model. Thus, the intra-frontal reductions observed in HbR in participants with elevated ASD traits are consistent with prior reports of decreased intra-prefrontal connectivity in individuals with ASD during cognitive tasks. In addition, the altered frontoparietal connectivity observed in HbR aligns with findings from previous resting-state studies highlighting network atypicality. Taken together, these results suggest that stratification based on AQ, rather than a categorical ASD diagnosis, may have utility and generalizability for identifying neurobiological variation associated with ASD traits. They further tentatively suggest that ASD traits comorbid with depressive symptoms may be characterized by network-level reorganization during VFT performance, marked by specific interhemispheric frontoparietal hyperconnectivity alongside diminished intra-frontal connectivity.

### 4.4 Potential stepwise shifts in micro- and macro-network reconfiguration

The results obtained in this study suggest not only FC abnormalities within each between-group comparison, but also a potential stepwise transition in network routing associated with the co-occurrence of depressive symptoms and autistic traits. Specifically, the combination of decreased interhemispheric frontal connectivity and localized HbR increases observed in Contrast 1 (Control vs. DP) shifted toward a prominent interhemispheric frontoparietal hyper-connectivity in Contrast 2, accompanied by concurrent intra-frontal connectivity reductions in HbR. This pattern indicates a possible progressive shift of prefrontal and frontoparietal network topology rather than a simple linear progression of disease-related connectivity deficits. Such suggestive variations in network configuration are consistent with the stepwise alterations in prefrontal function reported by Ohtani et al. (2021). The fact that these effects included reductions within the right prefrontal region in Contrast 2 also accords with reports of aberrant intra-right prefrontal connectivity in individuals with ASD during cognitive tasks (Han et al., 2022). Accordingly, a potential stepwise routing shift of prefrontal networks, involving localized right-prefrontal reductions and compensatory frontoparietal increases, may reflect a neural substrate specific to individuals with depressive symptoms and comorbid ASD traits.

### 4.5 Significance of dual-chromophore analyses

In this study, FC was analyzed based on both HbO_2_ and HbR signals. The HbR signal produces high theoretical (Buxton, 2012) and empirical (Huppert et al., 2006) consistency with BOLD responses. Furthermore, it has been pointed out that, in fNIRS, task-related systemic artifacts can produce false-positive or false-negative findings (Tachtsidis and Scholkmann, 2016), and that such artifacts are particularly pronounced in the HbO₂ signal (Kirilina et al., 2012; Abdalmalak et al., 2022). In contrast, HbO₂ is often argued to have higher sensitivity and better signal-to-noise ratio than HbR, and it is therefore more frequently reported in studies (Kinder et al., 2022). Similar to the recommendations in recent best-practice guidelines (Yücel et al., 2021) and in a review examining reporting practices for HbO_2_ and HbR (Kinder et al., 2022), the present study included analyses that do not rely on a single chromophore; thus, the results showing FC changes observed across both chromophores are more robust than those based on a single indicator. Although dual-chromophore analysis was performed, systemic physiological artifacts may still influence task-evoked FC estimates, particularly in HbO₂ signals.

### 4.6 Strengths

This study has several notable strengths. First, to our knowledge, this is the first study to examine task-evoked FC during a VFT in individuals with depressive symptoms stratified by ASD trait levels using fNIRS. This approach enabled the identification of neural network characteristics specific to the high-ASD-trait subgroup within a population with elevated depressive symptoms. Second, the use of dual chromophore analysis enhanced the robustness of the findings; the consistency of results across both signals strengthens confidence in the observed network abnormalities. Third, the adoption of effect size criteria (partial *η*^2^ ≥ 0.14) for determining meaningful group differences, rather than relying solely on *p*-values, provides a more clinically and scientifically interpretable framework for evaluating the magnitude of connectivity alterations. Such effect-size–based interpretation increases comparability across studies and facilitates the integration of evidence, including meta-analysis, thereby also contributing to cumulative clinical science (Cumming, 2014; Lakens et al., 2018). Fourth, clinical confounders such as depression severity, anxiety, and antidepressant and antipsychotic medication dosage were comparable between the AQ-high and AQ-low groups, reducing the likelihood that observed differences are attributable to these variables rather than ASD trait levels.

### 4.7 Limitations

Several limitations should be acknowledged when interpreting the present findings. First, ASD traits were assessed using the Autism-Spectrum Quotient (AQ), a self-report questionnaire, rather than formal diagnostic evaluation. Therefore, the high AQ group does not represent individuals with a clinical ASD diagnosis, and the results should be interpreted as reflecting dimensional variation in autistic traits within a depressed population rather than categorical ASD-depression comorbidity. Second, group differences were evaluated primarily based on effect size criteria (partial *η*^2^ ≥ 0.14 as “large”) rather than conventional significance testing with stringent alpha correction. While this approach emphasizes the magnitude and potential clinical relevance of the findings, it may include some false positives and should be replicated in independent samples. We should note that as consequence of the execution of mass-univariate edgewise analyses across multiple channel pairs, the possibility of false-positive findings remains. Third, the sample size, particularly for subgroup comparisons (AQ-high: *n* = 20; AQ-low: *n* = 23), was relatively modest, which may limit statistical power and the generalizability of the findings. Fourth, the cross-sectional design precludes causal inferences regarding the relationship between ASD traits and FC alterations in depression. Fifth, all participants were recruited from a single institution, which may limit the external validity of the findings. Despite these limitations, this study is important in clarifying neural network abnormalities in individuals with depressive symptoms and high ASD traits and demonstrating the potential of fNIRS as a candidate adjunctive diagnostic tool for the future. Taken together, the present findings should be interpreted within the framework of a clinically heterogeneous depressive-state cohort, reflecting real-world psychiatric populations rather than a single diagnostic entity.

## 5 Conclusion

In this study, we investigated task-evoked FC during the performance of a VFT in individuals experiencing depressive symptom, with a particular focus on the influence of comorbid autistic traits assessed using the AQ. Using fNIRS, we demonstrated that individuals experiencing depressive symptoms exhibited altered FC within prefrontal, fronto-temporal, and fronto-parietal networks, characterized by decreased interhemispheric frontal connectivity alongside network-specific increases and decreases, compared with neurotypical controls. Importantly, within the depression group, individuals with high AQ scores showed a network reorganization, marked by consistent increases in interhemispheric frontoparietal connectivity across both HbO₂ and HbR signals alongside localized intra-frontal reductions, indicating a complex pattern of network reconfiguration rather than a simple graded deficit associated with autistic traits. These findings extend previous evidence that both depression and autism spectrum conditions are characterized by large-scale network alterations and further highlight the relevance of task-evoked FC for capturing clinically meaningful neural alterations. In particular, the prominent involvement of interhemispheric frontoparietal and fronto-temporal networks observed in individuals with elevated autistic traits suggests altered integration between executive control and phonological monitoring or cognitive processing systems during cognitively demanding tasks. Such network-level alterations may contribute to the clinical complexity, treatment resistance, and social-cognitive difficulties frequently observed in depression with co-occurring autistic traits. From a methodological perspective, this study underscores the utility of combining a dimensional assessment of autistic traits with task-based fNIRS connectivity analysis. By including individuals with subthreshold depressive symptoms and stratifying participants based on AQ scores rather than categorical diagnoses alone, the present approach captures neurobiological heterogeneity that more closely reflects real-world clinical populations. The use of dual-chromophore analysis further strengthens the robustness of the observed connectivity patterns. Future studies with larger samples and longitudinal designs are warranted to determine whether these connectivity alterations represent stable trait markers or state-dependent changes associated with symptom severity and treatment response. Moreover, integrating fNIRS-based network measures with behavioral, clinical, and interventional data may facilitate the development of individualized assessment strategies and contribute to more precise, transdiagnostic approaches to mood and neurodevelopmental disorders. Overall, the present findings suggest that task-evoked fNIRS connectivity has potential as a candidate adjunctive marker for characterizing depression with autistic traits and for providing exploratory evidence of network-level alterations in psychiatric comorbidities.

## Data Availability

The raw data supporting the conclusions of this article will be made available by the authors, without undue reservation.
