# Supplementary material for: Reduced intra-frontal functional connectivity during a verbal fluency task in depressed individuals with autistic traits: an fNIRS study
Source: Front Hum Neurosci. 2026 Aug 27;20:1778517. doi: 10.3389/fnhum.2026.1778517 (PMC13558434; doi:10.3389/fnhum.2026.1778517)
Supplement: Supplementary file 1 [file Table_1.DOCX]

Supplementary Table S1

Supplementary Table S1. Diagnostic composition of the depressive-state cohort (n = 43)

Diagnosis category	n

---------------------------------------------------------------------------------------------

Major depressive disorder / recurrent depressive disorder	22

Bipolar affective disorder (depressive or mixed state)	17

Other depressive conditions (mainly dysthymic features)	4

---------------------------------------------------------------------------------------------

Total	43
