# Supplementary material for: Reduced intra-frontal functional connectivity during a verbal fluency task in depressed individuals with autistic traits: an fNIRS study
Source: Front Hum Neurosci. 2026 Aug 27;20:1778517. doi: 10.3389/fnhum.2026.1778517 (PMC13558434; doi:10.3389/fnhum.2026.1778517)
Supplement: Supplementary file 2 [file Table_2.DOCX]

Supplementary Table S2

Supplementary Table S2. Diagnostic composition by AQ group

Diagnosis category	AQ-high (n=20)	AQ-low (n=23)

----------------------------------------------------------------------------------------------------

MDD / recurrent depressive disorder	9	13

Bipolar affective disorder	10	7

Other depressive conditions	1	3

---------------------------------------------------------------------------------------------------

Total	20	23
